# The Leishmania donovani Ortholog of the Glycosylphosphatidylinositol Anchor Biosynthesis Cofactor PBN1 Is Essential for Host Infection

**DOI:** 10.1128/mbio.00433-22

**Published:** 2022-04-14

**Authors:** Adam Roberts, Rupa Nagar, Cordelia Brandt, Katherine Harcourt, Simon Clare, Michael A. J. Ferguson, Gavin J. Wright

**Affiliations:** a Cell Surface Signalling Laboratory, Wellcome Sanger Institute, Hinxton, Cambridge, United Kingdom; b Department of Biology, Hull York Medical School, York Biomedical Research Institute, University of York, York, United Kingdom; c Wellcome Centre for Anti-Infectives Research, School of Life Sciences, University of Dundee, Dundee, United Kingdom; d Pathogen Support Team, Wellcome Sanger Institute, Hinxton, Cambridge, United Kingdom; College of Veterinary Medicine, Cornell University

**Keywords:** glycophosphatidylinositol biosynthesis, parasitology, mass spectrometry, leishmaniasis, enzyme, bioluminescence

## Abstract

Visceral leishmaniasis is a deadly infectious disease caused by Leishmania donovani, a kinetoplastid parasite for which no licensed vaccine is available. To identify potential vaccine candidates, we systematically identified genes encoding putative cell surface and secreted proteins essential for parasite viability and host infection. We identified a protein encoded by *LdBPK_061160* which, when ablated, resulted in a remarkable increase in parasite adhesion to tissue culture flasks. Here, we show that this phenotype is caused by the loss of glycosylphosphatidylinositol (GPI)-anchored surface molecules and that *LdBPK_061160* encodes a noncatalytic component of the L. donovani GPI-mannosyltransferase I (GPI-MT I) complex. GPI-anchored surface molecules were rescued in the *LdBPK_061160* mutant by the ectopic expression of both human genes *PIG-X* and *PIG-M*, but neither gene could complement the phenotype alone. From further sequence comparisons, we conclude that *LdBPK_061160* is the functional orthologue of yeast *PBN1* and mammalian *PIG-X*, which encode the noncatalytic subunits of their respective GPI-MT I complexes, and we assign *LdBPK_061160* as *LdPBN1*. The *LdPBN1* mutants could not establish a visceral infection in mice, a phenotype that was rescued by constitutive expression of *LdPBN1*. Although mice infected with the null mutant did not develop an infection, exposure to these parasites provided significant protection against subsequent infection with a virulent strain. In summary, we have identified the orthologue of the PBN1/PIG-X noncatalytic subunit of GPI-MT I in trypanosomatids, shown that it is essential for infection in a murine model of visceral leishmaniasis, and demonstrated that the *LdPBN1* mutant shows promise for the development of an attenuated live vaccine.

## INTRODUCTION

*Leishmania* sp. parasites are estimated to infect up to 1 million individuals every year, with the vast majority of infections concentrated in low- and middle-income countries (1). Clinically, human leishmaniasis presents itself in either cutaneous, mucocutaneous, or visceral form, with the cutaneous form being responsible for the majority of cases and the more deadly visceral form having a fatality rate of up to 7% in areas where the disease is endemic (2). Leishmaniasis is currently treated with drugs that include amphotericin B, miltefosine, and antimonials; however, these drugs exhibit toxicity and teratogenicity, and the associated side effects make compliance with effective dosing regimens difficult (3). Importantly, there are reports that the parasite has evolved resistance to these drugs, leading to increased incidences of treatment failure (4–6). While drug screening and repurposing strategies are providing a new source of safe and effective antileishmanial compounds for assessment in the clinic (7–9), the need to continually treat new infections and the poor patient access to medical infrastructures in the regions of the world where leishmaniasis is endemic mean that an effective vaccine would be a valuable control tool for this disease.

Because of their direct accessibility to the host humoral immune response, proteins that are displayed on the surface of pathogens are often excellent vaccine candidates. One way that eukaryotic organisms attach proteins to the plasma membrane is to covalently couple polypeptides to a glycosylphosphatidylinositol (GPI) anchor. GPI anchors are attached to the C-terminal amino acid α-carboxyl group by an amide bond to the ethanolamine (EtN) residue of a conserved GPI anchor core structure of EtN-*P*-6Manα1-2Manα1-6Manα1-4GlcNα1-6*myo*-inositol-1-*P*-lipid, where the lipid can be phosphatidylinositol (PI) or inositol phosphoceramide (IPC). The conserved GPI anchor core structure can be substituted with sugar and nonsugar substituents in a species- and/or cell type-specific manner. The PI or IPC component is embedded in the outer leaflet of the plasma membrane, thus affording stable membrane association (10, 11). The synthesis of the core structure begins by the transfer of an α-linked *N*-acetylglucosamine (GlcNAc) to the inositol ring on the cytoplasmic side of the endoplasmic reticulum (ER) followed by de-N-acetylation to create GlcN-PI. This molecule is then “flipped” into the lumenal-facing leaflet of the ER membrane, where three α-linked mannose residues are added by the sequential activity of three distinct GPI mannosyltransferases (GPI-MT-I, -II, and -III) using dolichol-phosphate mannose as the donor substrate (10, 11). Following further processing, including the addition of the EtN-*P* group from the phospholipid phosphatidylethanolamine (PE), the GPI anchor replaces a C-terminal hydrophobic polypeptide sequence on the target protein in a transamidase reaction catalyzed in the ER by a GPI-transamidase enzyme complex (11).

The cell surfaces of parasitic protozoa, and especially kinetoplastids, are characterized by highly abundant proteins that are attached to the plasma membrane by GPI anchors and/or by GPI anchor-like glycoinositolphospholipids (GIPLs) and/or large (phospho)oligosaccharides attached to GPI anchor-like structures (10). The structural hallmark of all GPI and GPI-like structures is the motif Manα1-4GlcNα1-6*myo*-inositol-1-*P*-lipid, and type 1 GPIs include the protein GPI anchors and are defined by the sequence Manα1-6Manα1-4GlcNα1-6*myo*-inositol-1-*P*-lipid, whereas some GIPLs are type 2 GPIs with Manα1-3Manα1-4GlcNα1-6*myo*-inositol-1-*P*-lipid sequences [or hybrid GPIs with branched (Manα1-3)Manα1-6Manα1-4GlcNα1-6*myo*-inositol-1-*P*-lipid sequences]. The large (phospho)oligosaccharide containing structures are generally attached to type 2 GPI structures. Such molecules have long been associated with immune evasion, immune modulation, and host and vector cell interactions, and these include the highly abundant allelically expressed variant surface glycoprotein (VSG) of African trypanosomes (12, 13), the *Leishmania* spp. lipophosphoglycans (LPGs) (14), and the GP63 surface protease of *Leishmania* sp. parasites (15).

Several independent studies have demonstrated that GPI-anchored molecules are important virulence factors or required for transmission (16, 17). The biosynthetic pathway for protein GPI anchors was largely delineated in protists using cell-free lysates of Trypanosoma brucei (18) and forward genetics in Saccharomyces cerevisiae (19); in mammals, many enzymes were discovered by expression complementation cloning in chemically induced mutant CHO cells, for example (20, 21). The variations leading to the type II and hybrid GPI structures were determined in *Leishmania* spp. (10). While the core structure of the protein GPI anchor is well conserved between mammals and kinetoplastid parasites, there are differences in their assembly (11). For example, in contrast to mammals, in trypanosomes, the first mannose is added to GlcN-PI, which lacks inositol acylation (20, 22). These differences have the potential to be exploited to develop new antiparasitic drugs (23, 24) and also mean that identifying the direct functional orthologs for some of the biosynthetic enzymes based on sequence identity alone can be difficult. One example of this is the protein orthologous to mammalian PIG-X/yeast PBN1 (25, 26), which acts as a cofactor for the catalytic subunit of GPI mannosyltransferase 1 (PIG-M/GPI14) and which has so far eluded identification in kinetoplastid parasites by sequence similarity searching.

Here, we demonstrate that the protein encoded by the gene *LdBPK_061160* is the PIG-X/PBN1 functional homolog of the trypanosomatids. We show that while parasites lacking this protein are viable *in vitro*, it is required for patent host infection.

## RESULTS

### Parasites lacking LdBPK_061160 have an increased adhesion phenotype due to the loss of GPI-anchored surface molecules.

**FIG 1:**
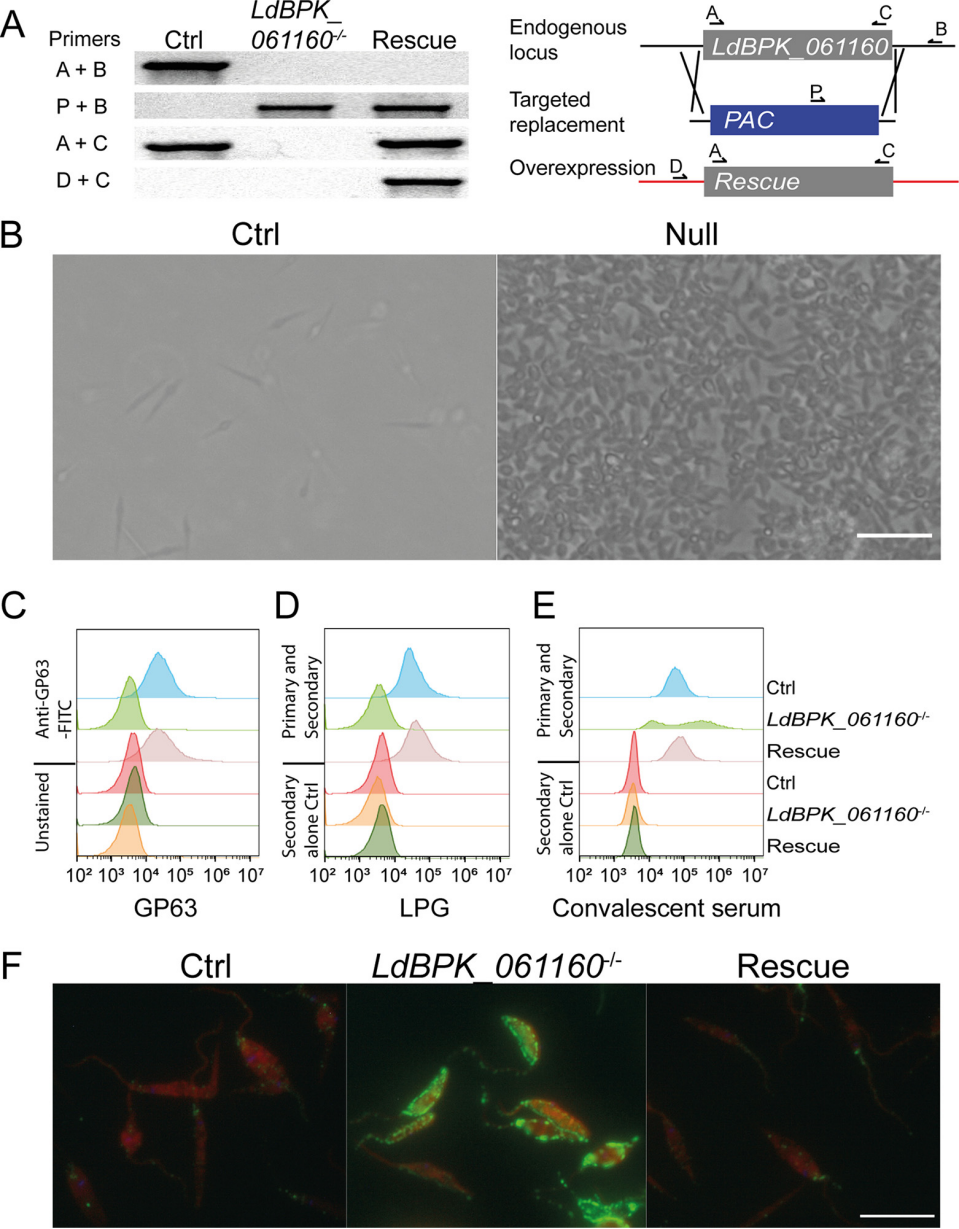
Genetic targeting of *LdBPK_061160* caused a cellular adhesion phenotype due to the catastrophic loss of cell surface GPI-anchored molecules. (A) Diagnostic PCRs demonstrating genetic targeting of *LdBPK_061160* locus. Schematic of the endogenous locus showing location and orientations of diagnostic primers and PCR products which demonstrate targeted replacement and ectopic overexpression of *LdBPK_061160* in the null mutant with genetic rescue from a nonendogenous locus. (B) Procyclic promastigote stage L. donovani parasites lacking *LdBPK_061160* exhibit increased adherence to tissue culture flasks (right) compared to the parental control (left). Bar, 20 μm. (C and D) Mutant parasites showed complete loss of cell surface GPI-anchored molecules as measured by flow cytometry, including GP63 (C) and LPG (D), compared to the parental control (Ctrl). In both panels C and D, surface expression was fully rescued by overexpression of *LdBPK_061160* (Rescue). (E) Parasites lacking *LdBPK_061160* were more brightly stained with convalescent-phase serum from chronically infected mice than the parental control, an effect that was rescued by expression of *LdBPK_061160*. (F) Images of the parasites analyzed for panel E showing that the epitopes recognized by chronically infected serum were distributed over the entire cell surface of the promastigote-stage L. donovani parasites. A representative image from two independent experiments is shown. Bar, 15 μm.

By systematically targeting genes encoding predicted cell surface and secreted proteins in Leishmania donovani using CRISPR/Cas9 technology, we previously identified a gene encoding a typical type I membrane protein, LdBPK_061160, which, when disrupted, caused a striking increase of parasite adhesion to the tissue culture flask plastic (Fig. 1A and B). It is known that the surface of procyclic promastigote stage *Leishmania* spp. parasites are dominated by GPI-linked molecules, including LPG, GIPLs and a protease (GP63) (14, 27), and so we asked if there were any major changes in the surface abundance of these molecules. By staining parasites with specific antibodies, we demonstrated complete ablation of LPG and GP63 on the surface of mutant parasites compared to the parental strain (Fig. 1C and D). Importantly, correct localization could be fully restored by constitutively expressing *LdBPK_061160*, demonstrating that this effect was specific to this gene and not attributable to off-target Cas9 activity (Fig. 1C and D). It has been suggested that these abundant parasite surface molecules may act as a steric shield, preventing the binding of host proteins, including antibodies, to the cell surface (28). Indeed, by contrast to the parental line, we observed that mutant parasites were brightly stained with antibodies present in convalescent-phase serum from chronically infected mice, confirming the loss of a protective glycocalyx (Fig. 1E). This increased antibody staining observed in the mutant was distributed across the whole cell surface, rather than being restricted to a particular region (Fig. 1F). Together, these data demonstrate an important role for *LdBPK_061160* in the presentation of GPI-anchored molecules at the surface.

### Loss of *LdBPK_061160* leads to the accumulation of GlcN-PI, the substrate of the GPI-MT I complex.

**FIG 2:**
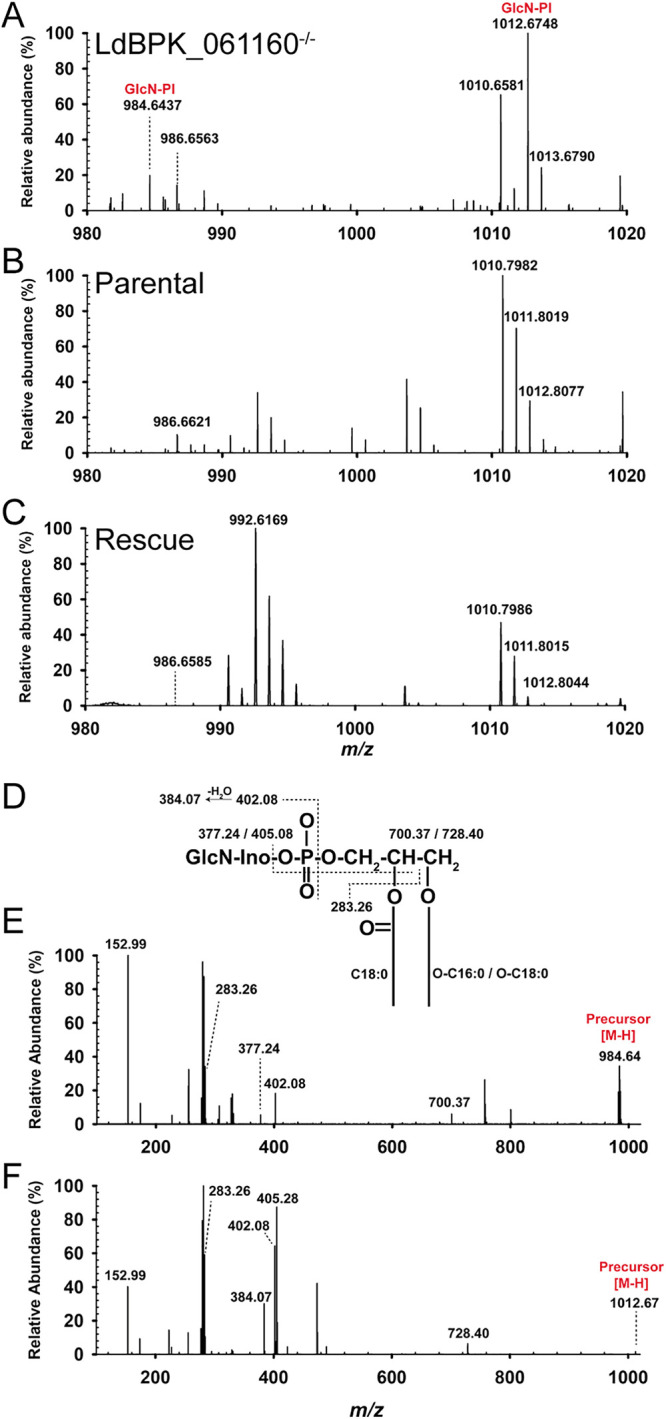
Disruptive targeting of *LdBPK_061160* causes an accumulation of the metabolite GlcN-PI demonstrating its requirement for GPI-mannosyltransferase I activity. Negative-ion electrospray mass spectrometry (ES-MS) spectra of lipid extracts from LdBPK_061160^−/−^ (A), parental strain (B), and rescued null (C) showing the selective accumulation of the GlcN-PI precursor ions (red). (D) Schematic showing ES-MS^2^ product ions of the GlcN-PI precursor ions. The ES-MS^2^ HCD (high-energy C-trap dissociation) product ion spectra for the [M − H]^−^ precursor ion at *m/z* 984.64 (E) and at *m/z* 1,012.67 (F) are shown. The difference between the precursors identified at *m/z* 984.64 (E) and 1,012.67 (F) was due to the length of the alkyl chain attached at position *sn-1* containing either C_16:0_ (E) or C_18:0_ (F). The product ion assignments for GlcN-PI species are indicated in panel D. The ion at *m/z* 402.08 representing [GlcN-*myo*-inositol-1,2-cyclic phosphate]^−^ and its dehydration product at *m/z* 384.07 are characteristic of negative GlcN-PI product ion spectra. The ions at *m/z* 152.99 and 283.26 are [glycerol-cyclic phosphate]^−^ and [CH_3_(CH_2_)_16_COO]^−^, respectively. The neutral loss of C_18:0_/(CH_3_(CH_2_)_16_COO)^−^ is represented by ions at *m/z* 700.37 (E) and 728.40 (F), whereas the neutral loss of C_18:0_/(CH_3_(CH_2_)_16_COO)^−^ and GlcN-inositol is represented by ions at *m/z* 377.24 (E) and 405.28 (F). The complete lipid profiles observed in negative-ion mode are shown in Fig. S1.

To characterize the function of *LdBPK_06116*0 in more detail, we first hypothesized that it may have a role in GPI anchor biosynthesis. To establish this, the lipid extracts from the parental, *LdBPK_06116*0^−/−^ mutant, and genetically rescued parasites were analyzed using negative-ion electrospray mass spectrometry (ES-MS) and molecular species confirmed by mass spectrometry of fragment ions (ES-MS^2^). The ES-MS data revealed the accumulation of a molecular species consistent with GlcN-PI in the *LdBPK_061160* null mutant, [M − H]^−^ ions at *m/z* 984.64 and *m/z* 1,012.67 (Fig. 2A). By contrast, the parental and genetically rescued cell lines both showed undetectable levels of these putative GlcN-PI species (Fig. 2B and C). The identities of the two putative GlcN-PI species were confirmed by ES-MS^2^ (Fig. 2D to F), with the observed difference in precursor masses a result of varying alkyl chain length at the *sn-1* position (C_16:0_ and C_18:0_ for *m/z* 984.64 and *m/z* 1,012.67, respectively). At the *sn-2* position, both precursors were found to have the same acyl chain (stearic acid, C_18:0_). Upon higher-energy C-trap dissociation (HCD) fragmentation in MS^2^, both [M − H]^−^ ions at *m/z* 984.64 and 1,012.67 produced an intense ion at *m/z* 402.08 (Fig. 2D to F) which corresponded to GlcN-*myo*-inositol-1,2-cyclic phosphate which is a characteristic negative product ion for GlcN-PI in ES-MS^2^. The alkylacyl based PI lipid species identified here are comparable to the previously reported GPI anchor lipid compositions in *Leishmania* (29–32). Because GlcN-PI species were undetectable in parental cells and mutant cells constitutively overexpressing *LdBPK_061160*, we conclude that the accumulation of GlcN-PI species in the mutant is caused by absence of *LdBPK_061160*. These data are consistent with mutant parasites lacking either a functional GPI-mannosyltransferase I (GPI-MT-I) complex or a flippase enzyme that transfers GlcN-PI from the cytosolic-facing leaflet of the lipid bilayer to the exoplasmic leaflet of the ER where the GPI-MT-I enzyme complex is localized.

10.1128/mbio.00433-22.1 FIG S1 Lipid profiling by negative-ion ES-MS identifies the bulk cellular inositol-phospholipids and the GlcN-PI GPI anchor-biosynthetic intermediate. Intense [M − H]^−^ ions were observed in parental (A), genetically rescued (B), and *LdBPK_61160*-null mutant (C) lipid extracts for inositol phosphorylceramide (triangles), diacyl-PI (circles), and alkylacyl-PI (squares) species. The identities of the major ions are indicated in the inset key. The identities of these species were subsequently confirmed by ES-MS^2^ (data not shown). Download FIG S1, PDF file, 0.2 MB. Copyright © 2022 Roberts et al. 2022 Roberts et al. https://creativecommons.org/licenses/by/4.0/ This content is distributed under the terms of the Creative Commons Attribution 4.0 International license.

The negative-ion ES-MS also identified the abundances of other major lipid species, including inositol phosphorylceramide (IPC), and phosphatidylinositol (PI) lipids, which were relatively unchanged in *LdBPK_06116*0^−/−^ parasites compared to the parental and genetically rescued lines (Fig. S1). In ES-MS^2^, IPC and PI-based lipid species produced strong product ions at *m/z* 241 and *m/z* 259 corresponding to inositol-1,2-cyclic phosphate and inositol-monophosphate which are characteristic product ions of IPC and PI-based lipids in negative-ion mass spectrometry (data not shown). The lipid species identified here corroborate with previously reported lipid species in *Leishmania* (33, 34).

### *LdBPK_061160* is the *PBN1* orthologue in L. donovani.

**FIG 3:**
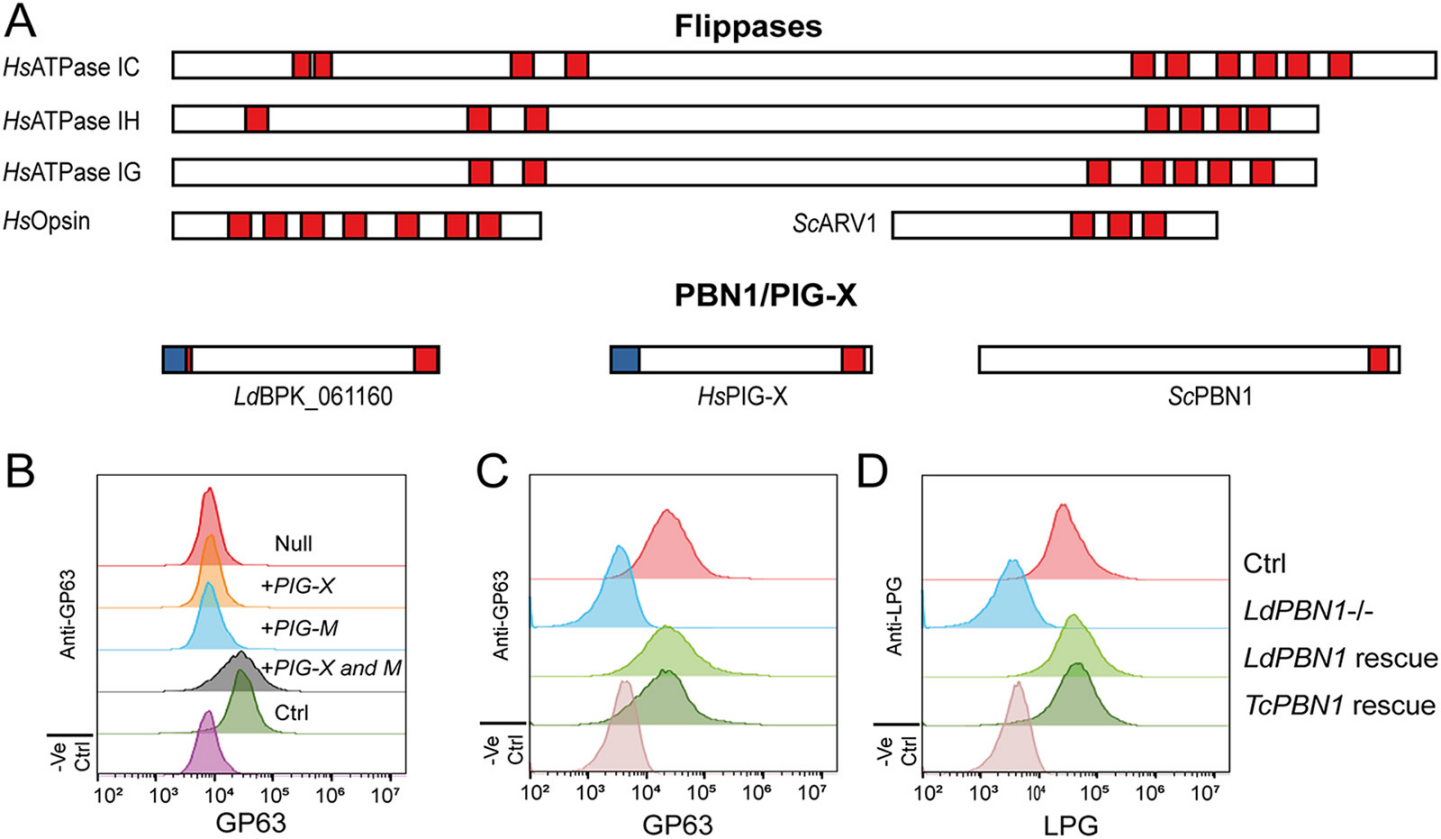
LdBPK_061160 is the L. donovani PBN1 orthologue. (A) Comparison of the protein sequence architecture of LdBPK_061160 with proteins of known flippase activity and the human and yeast PIG-X/PBN1 proteins. Note that LdBPK_061160 shares structural features with the PIG-X/PBN1 proteins and not the flippases. Predicted signal peptides are in blue, and transmembrane regions are in red. (B) Overexpression of the genes encoding Homo sapiens PIG-X and PIG-M in LdBPK_061160 mutants restores presence of GPI-anchored proteins at the cell surface. Histograms show cell surface staining of the GPI-anchored GP63 protein in LdBPK_061160 mutants overexpressing human genes encoding PIG-X, PIG-M, or both using a FITC-conjugated anti-GP63 monoclonal antibody by fluorescence-activated cell sorting (FACS). (C and D) Experimental identification of the PBN1 orthologue in T. cruzi. LdPBN1 mutants that lack surface expression of the GPI-anchored cell surface molecules GP63 and LPG were compared to the parental line and were genetically complemented with the gene encoding T. cruzi PBN1 (*TcPBN1*) (Contig ADWP02007247; nucleotides 5233.6071) or *LdPBN1* as a positive control. Levels of cell surface GP63 (C) and LPG (D) were quantified by staining parasites with monoclonal antibodies and flow cytometry. Results of one representative experiment of three are shown.

The mass spectrometry data alone were unable to distinguish whether *LdBPK_061160* functioned as an ER-localized flippase, or a component of the GPI-MT I complex. To distinguish between these possibilities, we first analyzed the structural architectures of the two classes of protein. While the identity of the ER-localized phospholipid flippase responsible for transferring GlcN-PI from the cytosolic face of the ER to the luminal face has yet to be elucidated (even with the advances in genome-wide CRISPR knockout screens [35]), there have been multiple enzymes identified from other organelles and organisms with reported phospholipid flipping activity (36–39). Sequence analysis of these phospholipid flipping enzymes reveal that they contain multiple (typically 4 to 10) transmembrane-spanning regions, whereas the protein architectures of PBN1 and PIG-X are more typical of a type I membrane protein with an N-terminal signal peptide and a single C-terminal transmembrane-spanning region (Fig. 3A). Based on this analysis, we concluded that the protein architecture of LdBPK_061160 is not consistent with a flippase enzyme.

To experimentally demonstrate that LdBPK_061160 is a PBN1 homolog, we attempted to rescue the loss of cell surface GPI-anchored molecules in the mutant parasite by the ectopic overexpression of the well-characterized human GPI-MT I enzymes. In agreement with previous studies, overexpression of human *PIG-X* or *PIG-M* alone was not individually capable of restoring the functional GPI pathway in the *LdBPK_061160* mutant; however, coexpression of both PIG-X and PIG-M restored staining of the GPI-anchored protein GP63 to the promastigote cell surface (Fig. 3B). Together, these data confirm that *LdBPK_061160* functions as the Leishmania donovani PBN1 homolog, and we therefore renamed this gene *LdPBN1*, in keeping with naming traditions for the trypanosomatid parasites.

One of the reasons that LdBPK_61160 had not previously been identified as a PIG-X/PBN1 orthologue is that the orthologues from different species vary significantly in size and have only low levels of sequence similarity. Consequently, simple sequence conservation had failed to identify the PIG-X/PBN1 homolog present in the related parasites T. brucei and Trypanosoma cruzi, which are the etiological agents of sleeping sickness and Chagas’s disease, respectively. Because of greater sequence conservation among trypanosomatidae parasites, we were able to identify candidate PBN1 orthologues in all trypanosomatid species by sequence matching to LdBPK_61160 (Table S1 and Fig. S2). To establish whether these candidates were indeed functional orthologues, we asked whether the T. cruzi orthologue from the strain Silvio X10-7 (Contig ADWP02007247; nucleotides 5233.6071; 35% amino acid identity; referred to here as *TcPBN1*) could genetically complement the *LdPBN1* null mutant (Fig. S3). Overexpression of *TcPBN1* restored surface presentation of both GP63 and LPG to the same extent as the *LdPBN1* control, demonstrating that it is also a functional PBN1 (Fig. 3C and D).

10.1128/mbio.00433-22.2 FIG S2 Phylogenetic tree generated from a Clustal Omega alignment of selected PBN1 homologs from selected parasites. Numbers in red represent the percent amino acid identity between LdBPK_061160 and each species. Download FIG S2, PDF file, 0.3 MB. Copyright © 2022 Roberts et al. 2022 Roberts et al. https://creativecommons.org/licenses/by/4.0/ This content is distributed under the terms of the Creative Commons Attribution 4.0 International license.

10.1128/mbio.00433-22.3 FIG S3 Confirmation of *TcPBN1* rescue genotyping. (Left) Diagnostic PCRs demonstrating that the loss of *LdBPK_061160* is due to targeted replacement at the endogenous locus (primers A + B or P + B) and genetic reconstitution with either *LdBPK_061160* (primers A + C or D + C) or *TcPBN1* (primers E + F or D + F) is specific to a nonendogenous locus. (Right) Schematic of the *LdBPK_061160* endogenous locus and targeted replacement with *PAC* with the *TcPBN1* and *LdPBN1* genetic rescues with associated diagnostic primers. Download FIG S3, PDF file, 0.2 MB. Copyright © 2022 Roberts et al. 2022 Roberts et al. https://creativecommons.org/licenses/by/4.0/ This content is distributed under the terms of the Creative Commons Attribution 4.0 International license.

10.1128/mbio.00433-22.5 TABLE S1 Identification of PBN1 homologs in other trypanosomatid parasites. Percent identity at the amino acid level was calculated from a pairwise comparison of a Clustal Omega multiple-sequence alignment. Download Table S1, PDF file, 0.2 MB. Copyright © 2022 Roberts et al. 2022 Roberts et al. https://creativecommons.org/licenses/by/4.0/ This content is distributed under the terms of the Creative Commons Attribution 4.0 International license.

### LdPBN1 is essential for host infection.

**FIG 4:**
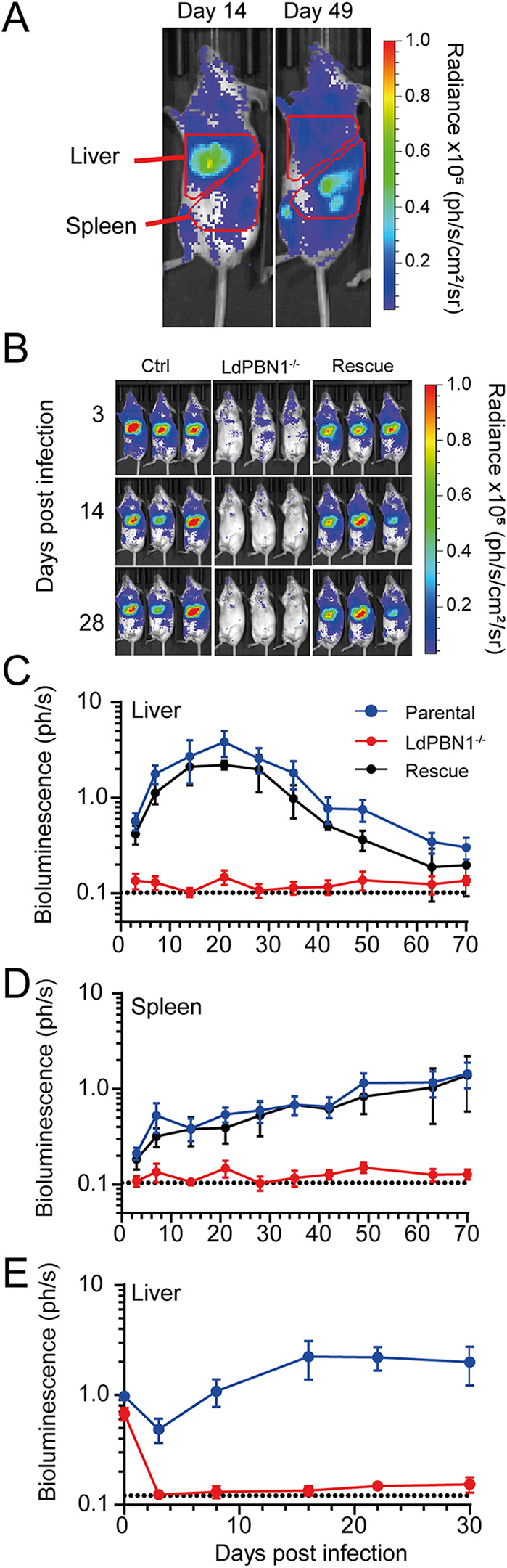
L. donovani PBN1 is essential for host infection. (A) Diagram showing examples of how infected animals were regionally gated to quantify infection load using bioluminescence imaging of the liver and the area corresponding to both the spleen and inguinal lymph node. (B) Groups of five mice were inoculated with stationary-phase promastigotes from parental (Ctrl), *PBN1^−/−^*, and *PBN1^−/−^* parasites transfected with a *PBN1* expression rescue plasmid (rescue), and infections were longitudinally monitored by bioluminescence imaging. Quantification of liver (C) and spleen (D) bioluminescent signals from parental control parasites (blue), PBN1-deficient (red) and PBN1-deficient parasites rescued by overexpression of the PBN1 (black). The units of bioluminescence are 1 × 10^6^ photons per second. Data are means and standard deviations (SD) (*n *= 5). Dotted line represents the average background bioluminescence from five unchallenged mice from a separate experiment measured over 77 days. Quantification of PBN1^−/−^ infection between 4 h to 30 days postinfection in the liver (E). Bioluminescence is reported as 1 × 10^6^ photons per second, and the data are from a single experiment where the group size was 5.

We next asked whether LdPBN1 mutants, which are viable *in vitro*, were able to establish an infection in a mammalian host. We used the LV9 strain of L. donovani, which stably expresses the firefly luciferase transgene, which permitted the longitudinal quantification of *in vivo* infection parameters using bioluminescent imaging (40). Using this model, parasites initially infect the liver and multiply to a peak at 2 weeks postinfection before they are gradually cleared and the focus of infection subsequently shifts to the spleen (Fig. 4A). Mice were inoculated intravenously with stationary-phase promastigotes from the bioluminescent parental strain, an LdPBN1-null mutant, and “rescued” mutants that constitutively expressed ectopic *LdPBN1.* As expected, we observed that mice infected with the parental strain showed robust infections that localized to the liver as early as day 3 postinfection (Fig. 4B and C), and which gradually progressed to the spleen (Fig. 4D). By contrast, *LdPBN1^−/−^* mutants failed to establish any detectable infection, with mice exhibiting only background levels of bioluminescence after 3 days (Fig. 4B and C). This effect could be rescued in the mutants by exogenous *LdPBN1*, confirming the causality of this gene (Fig. 4B and C). The inability of the *LdPBN1^−/−^* mutant to infect mice was reproducible showing the presence of a bioluminescent signal detected in the liver at 4 h postinfection which was reduced to background levels by day 3 (Fig. 4E), confirming that the mice challenged with an infectious dose were capable of rapidly controlling the infection. To determine if mutant parasites could persist below the limits of detection, we attempted to recover parasites from the spleens of infected mice by culturing homogenates from isolated spleens. No *LdPBN1^−/−^* parasites were recovered despite readily recovering viable parasites from mice infected with parasites with a functional copy of *LdPBN1*, suggesting that GPI-anchored surface molecules are required for infection of mice.

### Exposure to *LdPBN1*-deficient parasites elicits protection against the development of visceral leishmaniasis.

**FIG 5:**
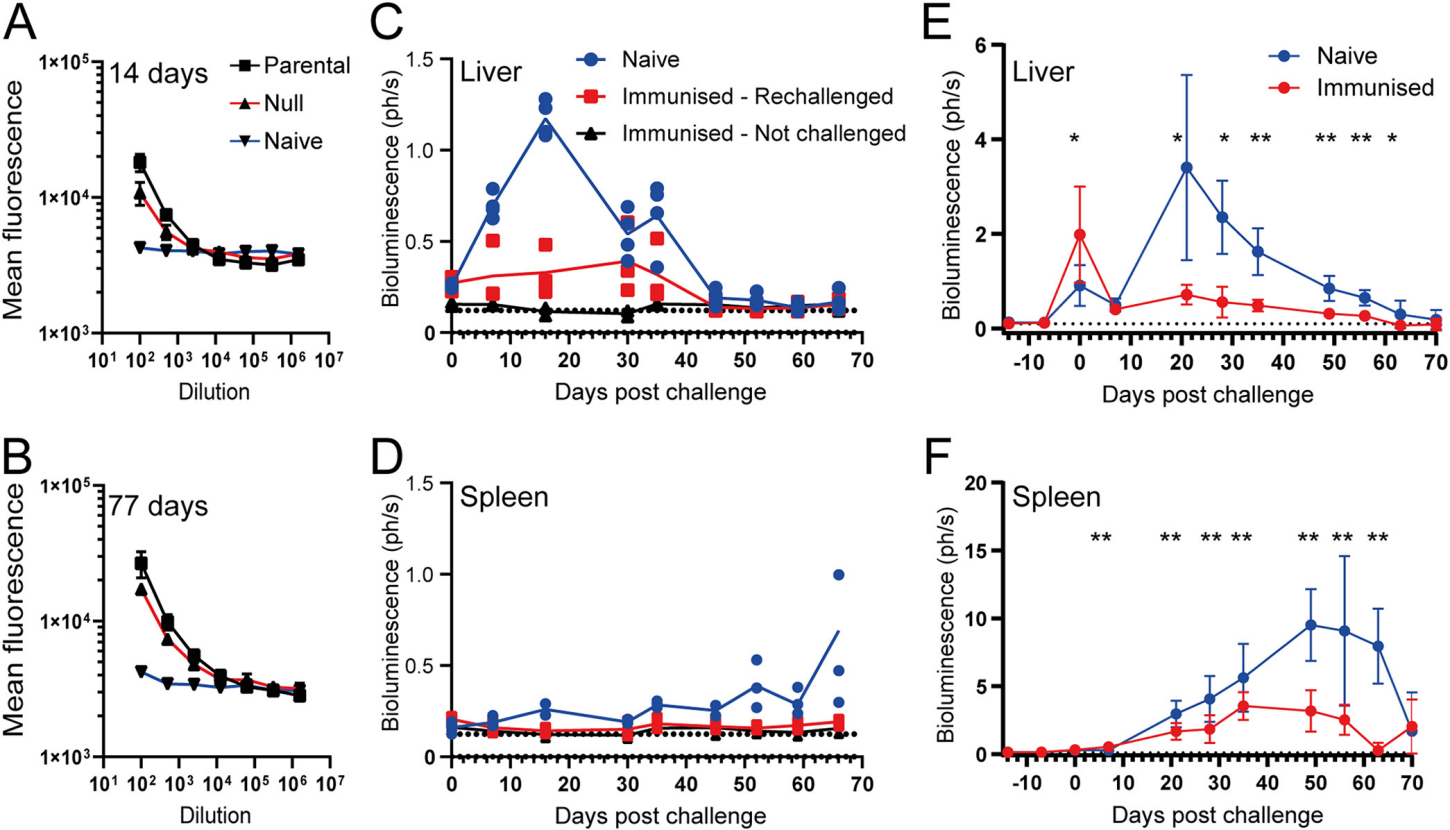
Inoculation with the LdPBN1 mutant is capable of eliciting a robust humoral immune response that offers significant protection against reinfection. Serum harvested from BALB/c mice that had been inoculated with either parental L. donovani parasites (black line) or PBN1^−/−^ parasites (red line) for 14 (A) and 77 (B) days was diluted and used to stain live promastigotes by fluorescence-activated cell sorting, and results were compared to those for control mouse sera (blue line). (C to F) Inoculation with PBN1^−/−^ parasites elicits protection against subsequent L. donovani infection. (C) Mice inoculated with the L. donovani PBN1^−/−^ mutant were rested for 84 days before being infected with the bioluminescent virulent parental L. donovani strain, and the parasitemia was quantified in the livers (C) and spleens (D) of infected animals (red line, *n *= 3). Two mice were left uninfected to control for the possibility of recrudescence (black line), and four naive mice were infected as a positive infection control (blue line). (E and F) A group of 10 mice were inoculated with PBN1^−/−^ mutant parasites and rested for 8 weeks prior to reinfection with the parental L. donovani strain and parasitemia quantified by bioluminescence in the liver (E) and spleen (F). Significantly different levels of parasitemia were assigned using a two-tailed Student's *t* test. *, *P* < 0.05, and **, *P* < 0.001, compared to a control group of 10 unimmunized mice. Data are from a single experiment, and bioluminescence is reported as 1 × 10^6^ photons per second.

Because the *LdPBN1^−/−^* mutant parasites were viable *in vitro* but incapable of establishing an infection *in vivo*, we asked whether they could be used as a genetically attenuated live parasite vaccine. We first determined if the mice inoculated with the *LdPBN1^−/−^* mutant parasites elicited a humoral immune response to the *LdPBN1^−/−^* mutant parasites by quantifying the ability of sera from infected mice to opsonize the cell surface of live promastigotes. Mice infected with either the parental L. donovani strain or the *LdPBN1^−/−^* mutant were able to generate a robust immune response at 14 days (Fig. 5A), with a further ∼1.5-fold increase observed at 77 days postinfection (Fig. 5B). Despite the rapid clearance of the parasite, the titer of antibodies elicited by the *LdPBN1^−/−^* mutant recognizing the parasite cell surface was remarkably only fractionally lower than the parental line (Fig. 5A and B).

We next investigated if this host-elicited immune response against parasites that lack GPI-anchored cell surface molecules was capable of preventing or controlling an infection with a virulent strain. A pilot group of five mice that had been inoculated with *LdPBN1^−/−^* mutant L. donovani parasites for 12 weeks were segregated into two groups. Three mice were infected with the virulent bioluminescent parental strain, while the remaining two mice were left unchallenged to monitor any possible recrudescence that would confound our analysis. We observed that the three mice that had been preexposed to *LdPBN1^−/−^* mutant parasites could control the peak in liver bioluminescence, and the infection did not progress to the spleen (Fig. 5C and D). These findings were replicated using a larger cohort of 10 mice but with a reduced time between the initial inoculation and challenge. We observed that although immunized mice had a higher liver burden than their nonimmunized counterparts at 4 h postinfection (Fig. 5E), possibly due to antibody-dependent enhancement of infection, they were able to robustly control the initial infection in the liver. The immunized mice also showed significant levels of protection in the spleen between 28 and 56 days before the naive mice naturally resolved their infection after 60 days (Fig. 5F). Together, these results confirm that mice exposed to L. donovani lacking major classes of cell surface molecules are able to elicit a protective effect against the uncontrolled development of visceral leishmaniasis.

## DISCUSSION

We have shown here that *LdBPK_061160* is the functional PBN1 homolog in Leishmania donovani parasites which is a necessary component of the glycosylphosphatidylinositol-mannosyltransferase I enzyme complex that performs an essential step in the GPI anchor-biosynthetic pathway. Although GPI anchors are present in all known eukaryotes, some components, including PIG-X/PBN1, show significant variation in sequence conservation, making them difficult to identify by sequence searching alone (25, 26).

Using functional complementation to restore GPI-MT I activity, we confirmed the role of *LdPBN1* by coexpression of both human *PIG-X* and *PIG-M* in LdBPK_061160-deficient parasites, and we were also able to identify, through sequence similarity, the PBN1 gene in T. cruzi and, by orthology, the same gene across all trypanosomatid species. This was recently confirmed by another group, who reported the identification and characterization of *Tb*PBN1 while revisions were being made to our manuscript (41). Remarkably, the sequence identity between L. donovani and T. cruzi PBN1 is just 35%, raising the question of what the minimal PBN1 sequence requirements are for stabilizing the GPI14 protein and complementing GPI-MT I function. It is known that the human and yeast gene products share only 19% amino acid identity and are functionally incompatible (Fig. S4), while the rat PIG-X homolog, which is 78% identical to the human protein, is capable of supporting the growth of yeast expressing *Hs*PIG-M, albeit at a significantly reduced rate (21, 26). While this confirms that cross-species complementation of function is possible, there cannot be too much phylogenetic separation, because the orthologue of GPI14 from Plasmodium falciparum or T. brucei was incapable of restoring growth in GPI14-disrupted yeast (26). The inability of orthologues to complement loss of function across species is not uncommon for proteins where the sequence similarity is low. Analysis of human genes able to complement the loss of essential yeast genes reveals that they are typically shorter and have a higher sequence identity (42). In addition, they were less likely to be a component of a macromolecular complex and predicted to have fewer physical interactions. These observations could generally explain the limited ability to rescue of GPI-MT I activity through overexpression of homologs from more distantly separated ancestors. One possibility is that the functional context of human PIG-X differs from that of LdPBN1, and indeed, PIG-X is known to interact with two other proteins, RCN1 and RCN2 (43).

10.1128/mbio.00433-22.4 FIG S4 Alignment of *ScPBN1*, *LdBPK_061160*, and *HsPIG-X.* Alignment was generated using Clustal Omega showing conserved amino acids (*) between all three orthologues. Download FIG S4, PDF file, 0.3 MB. Copyright © 2022 Roberts et al. 2022 Roberts et al. https://creativecommons.org/licenses/by/4.0/ This content is distributed under the terms of the Creative Commons Attribution 4.0 International license.

To demonstrate that *LdBPK_061160* was required for activity of the GPI-MT I complex, we showed that lipid extracts from the mutant parasites accumulated GlcN-PI, the precursor substrate for GPI-MT I. Interestingly, in comparison to the lipid extracts analyzed from T. brucei lacking the enzyme from the preceding step in the biosynthetic pathway (GlcNAc-PI de-N-acetylase, which is encoded by GPI12 and which converts GlcNAc-PI to GlcN-PI), a much smaller amount of the precursor lipid appears to accumulate (16). This could be an artifact in the extraction or analysis, or it could be that GlcN-PI exerts product inhibition on the GlcNAc-PI de-N-acetylase, limiting the accumulation of GlcN-PI, or that GlcN-PI is rapidly metabolized/catabolized in the absence of GPI-MT I activity.

Our initial interest in *LdBPK_061160* was as a possible cell surface vaccine target because it has primary sequence features that are compatible with it being an antibody-accessible cell surface membrane protein; however, our findings demonstrate that it is more likely to be localized to the intracellular endoplasmic reticulum membrane, where GPI-MT I activity is localized (44). It may, however, be possible to interfere with the interaction between PBN1 and GPI14 using rationally designed small molecules (45). We have shown that inhibiting the GPI biosynthesis pathway in L. donovani would affect parasite viability *in vivo*, and the significant protein sequence diversity between the parasite and mammalian proteins suggests that a drug could be designed that is specific for the parasite and lacks unwanted toxicity. This would be facilitated by characterizing the molecular details of the GPI14/PBN1 interaction interface. It is known that in the absence of PIG-X, the abundance of the catalytic subunit PIG-M is greatly reduced, indicating that the role of PBN1/PIG-X is as a stabilizing cofactor and/or molecular chaperone (21). This conclusion is further supported by an observed increase in the unfolded protein response in yeast in the absence of PBN1 (46), and chaperones have already been identified to be potential drug targets for diseases caused by protozoan parasites (47, 48).

The functional consequences of disrupting GPI-MT I in L. donovani are comparable with studies characterizing other mutants of the GPI anchor-biosynthetic enzymes in related parasites, including the observed increased adhesion phenotype (16, 17, 49, 50). Also, assessment of genes involved in mannose activation in Leishmania mexicana generally shows a requirement for phosphomannose mutase (51), GDP-mannose pyrophosphorylase (50, 52), for virulence, but not dolicholphosphate-mannose synthase (51). This is in contrast to our observations that the absence of multiple GPI-anchored surface molecule classes appears to affect the virulence of the parasites *in vivo*, as individually, parasites with targeted loss of LPG (53) or GPI-anchored proteins (54) are still able to infect mice. In the related parasite T. brucei, GPI-anchored proteins are necessary for cell survival in the bloodstream but not procyclic forms (55), and the underlying reason for this difference has yet to be fully elucidated, but it could plausibly be due to loss of the transferrin receptor, which is GPI anchored in T. brucei and essential for viability of bloodstream-form parasites (23, 56). It may be possible to address this by creating a non-GPI-anchored form of the transferrin receptor (57). Procyclic forms of T. brucei are thought to acquire iron from ferric complexes via a reductive mechanism (58), and consistent with this, *Leishmania* parasites acquire iron from their medium via a non-GPI anchor-mediated mechanism (59, 60), possibly explaining the discrepancies in observed lethalities between these parasites.

Finally, there is evidence that this mutant may have the potential to be exploited as a genetically attenuated live vaccine due to the significant levels of protection observed in both the liver and spleen. However, further investigations are needed to determine if this would be protective in the hamster model (61) or, alternatively, if this mutant would also provide protection against cutaneous disease (62). Despite a strong humoral response elicited by infection with the mutant, it remains to be determined if parasites lacking major components of its glycocalyx are capable of eliciting antibodies that recognize a broader range of surface proteins or protein epitopes. Our study adds another potential candidate to the list of candidates for an attenuated vaccine for the treatment of leishmaniasis, which currently includes the biopterin transporter (63), KHARON1 (64), p27 (65), and centrin. The centrin-null mutant has demonstrated protection against infection with a cutaneous-illness-causing strain (66) in addition to eliciting a protective response when it is replaced in a visceral-illness-causing species (67). Our findings here could therefore contribute to the development of a novel vaccine that could help limit or reduce the impact of this deadly infectious parasitic disease.

## MATERIALS AND METHODS

### Ethics statement.

All animal experiments were performed under United Kingdom Home Office regulations (license numbers P98FFE489 and PD3DA8D1F) and European directive 2010/63/EU. Research was ethically approved by the local Sanger Institute Animal Welfare and Ethical Review Board. Mice were maintained under a 12-h light/dark cycle at a temperature of 19 to 24°C and humidity between 40% and 65%. The animals used were 6- to 8-week-old female Mus musculus strain BALB/c obtained from a breeding colony at the Wellcome Sanger Institute.

### L. donovani cell culture.

Parasites were maintained as described previously (40) in continuous culture supplemented with or without nourseothricin, hygromycin, blasticidin, puromycin, or phleomycin at 100, 50, 25, 15, and 20 μg mL^−1^ as appropriate. Single-cell suspensions were obtained by detaching parasites through vigorous shaking and passing through a 10-μm cell strainer (pluriStrainer).

### CRISPR-Cas9-mediated deletion of *LdBPK_061160* and genetic rescue.

The *LdBPK_061160* gene was targeted by CRISPR-Cas9 mediated deletion using the approach previously described (68) with the primers documented in Table 1, and correct targeting by CRISPR-Cas9 was assessed by PCR. The open reading frames (ORFs) of *LdBPK_061160* and T. cruzi were amplified using primers designed against *TcCLB.508173.240* from T. cruzi Silvio X10/7 genomic DNA, with primers containing engineered NheI and XhoI endonuclease restriction sites (Table 1). The purified PCR products and the plasmid PTBLE were digested with NheI and XhoI and the ORFs ligated into the linearized plasmid yielding PTBLE-*LdBK_061160* and PTBLE-*TcPBN1,* respectively. SwaI-digested plasmids were electroporated into mid-log-phase promastigotes using an Amaxa Nucleofector 2b device as previously described (68). Transgenic parasites were selected by the addition of phleomycin (20 μg mL^−1^) until parasites in a no-plasmid control transfection had died. cDNAs encoding *HsPIG-X* and *HsPIG-M* were amplified using specific primers (Table 1) from plasmids carrying genes NM_017861.3 and NM_145167.2 (Sino Biological), and the PCR products were cloned into PTBLE and pRIB expression plasmids using standard restriction enzyme methods (69).

**TABLE 1:** Primers used to amplify ORFs for overexpression

Primer	Sequence
*LdBPK_061160*_F	CTAGGCTAGCATGTCGCGCGTAGTCGTGATG
*LdBPK_061160*_R	CTACTCTCGAGCTAACGAATAGCGAGGATAACGA
*TcPBN1*_F	CTAGGCTAGCATGATGCCTCTTTGTGTTTCTCC
*TcPBN1*_R	CTACTCTCGAGCTAAACACGCAGCAGCGAAA
*HsPIGX*_F	ATCTTAGCTAGCATGGCGGCTCGGGTGGCG
*HsPIGX*_R	ATCTTACTCGAGTTATAGGGAAAAATGGCCATATTTGAAAACTGCTACAAGGATCAATGTAG
*HsPIGM*_F	AATCACTAGATCTCTCGAGATGGGCTCCACCAAGCACTGGGG
*HsPIGM*_R	ATCTTAAGATCTCTAGTCATATTTGATTCTCTCTGTCAGGGGTTCTTCTTTGTAATGG

### Mouse infections and serum collection.

Groups of five female BALB/c mice were infected with 1 × 10^8^ stationary-phase promastigotes via the intravenous route as previously described (40). Serum was collected from infected mice through tail bleed and clotted by incubating at 37°C for 30 min, prior to centrifugation at 20,000 × *g* for 15 min.

### Flow cytometry and microscopy.

Mid-log promastigotes were harvested by centrifugation at 800 × *g* for 5 min at 23°C and resuspended at a concentration of 1.1 × 10^8^ mL^−1^ in ice-cold phosphate-buffered saline (PBS). A total of 1 × 10^7^ parasites were stained with or without mouse anti-GP63–FITC (fluorescein isothiocyanate) (clone 96/26, used at a 1/100 dilution), mouse anti-LPG (clone CA7AE, used at a 1/10,000 dilution), or mouse anti-L. donovani (polyclonal, used at a 1/1,000 dilution) for 1 h at 4°C. Parasites were washed three times in PBS and stained with goat anti-mouse IgG–Alexa Fluor 488 (1/1,000 dilution), goat anti-mouse IgG–Alexa Fluor 633 (1/1,000 dilution), or rabbit anti-IgM–Alexa Fluor 633 (1/1,000 dilution) for 1 h at 4°C prior to washing in PBS three times. Washed parasites were biologically inactivated with a 2% formalin solution buffered in PBS for 15 min at 23°C, and buffer was exchanged into PBS for analysis on a CytoFLEX S flow cytometer (Beckman Coulter). A total of 10,000 events were collected and analyzed in FlowJo (v10.6.1). Fluorescent images were acquired on an Axio Vert.A1 equipped with an AxioCam ERc 5s, with a Colibri 7 LED light source using appropriate filter sets and a 40× or 100× objective. Exposure times were standardized and the monochromatic images processed in Fiji (70).

### Metabolite identification from lipid extracts by mass spectrometry.

Promastigotes of parental, *LdBPK_061160* null mutant and genetically rescued cells of L. donovani (5 × 10^8^ of each) in mid-logarithmic growth were harvested by centrifugation and washed three times with PBS. The lipids were extracted as previously reported (16). Briefly, the cells were extracted with chloroform-methanol-water (10:10:3 [vol/vol/vol]) overnight at 4°C and sonicated for 15 min in a sonicating water bath. The extract was centrifuged for 5 min at full speed, and the supernatant was dried under a nitrogen stream in a fresh tube. The dried extract was subjected to butan-1-ol–water (1:1 [vol/vol], 200 μL each) partition, and the aqueous phase was partitioned twice with 0.2 mL water-saturated butan-1-ol. The butan-1-ol phases were pooled and backwashed three times with 0.4 mL water saturated with butan-1-ol. The washed butan-1-ol phases were dried under a nitrogen stream and dissolved in 50 μL chloroform-methanol-water (10:10:3) for electrospray-ionization mass spectrometry (ES-MS) analysis. Five microliters of sample was infused into the Orbitrap Fusion Tribrid mass spectrometer (Thermo Scientific) using static infusion nanoflow probe tips (M956232AD1-S; Waters). ES-MS and ES-MS^2^ analysis were performed in negative-ion mode using negative-ion spray voltage as 0.7 kV and the ion transfer tube temperature was 275°C. Collision-induced dissociation (CID) and high-energy C-trap dissociation (HCD) were used for ES-MS^2^ fragmentation, using 30 to 45% collision energy.
