# Supplementary material for: The Leishmania donovani Ortholog of the Glycosylphosphatidylinositol Anchor Biosynthesis Cofactor PBN1 Is Essential for Host Infection
Source: mBio. 2022 Apr 14;13(3):e00433-22. doi: 10.1128/mbio.00433-22 (PMC9239262; doi:10.1128/mbio.00433-22)
Supplement: FIG S1 [file mbio.00433-22-s0002.pdf]

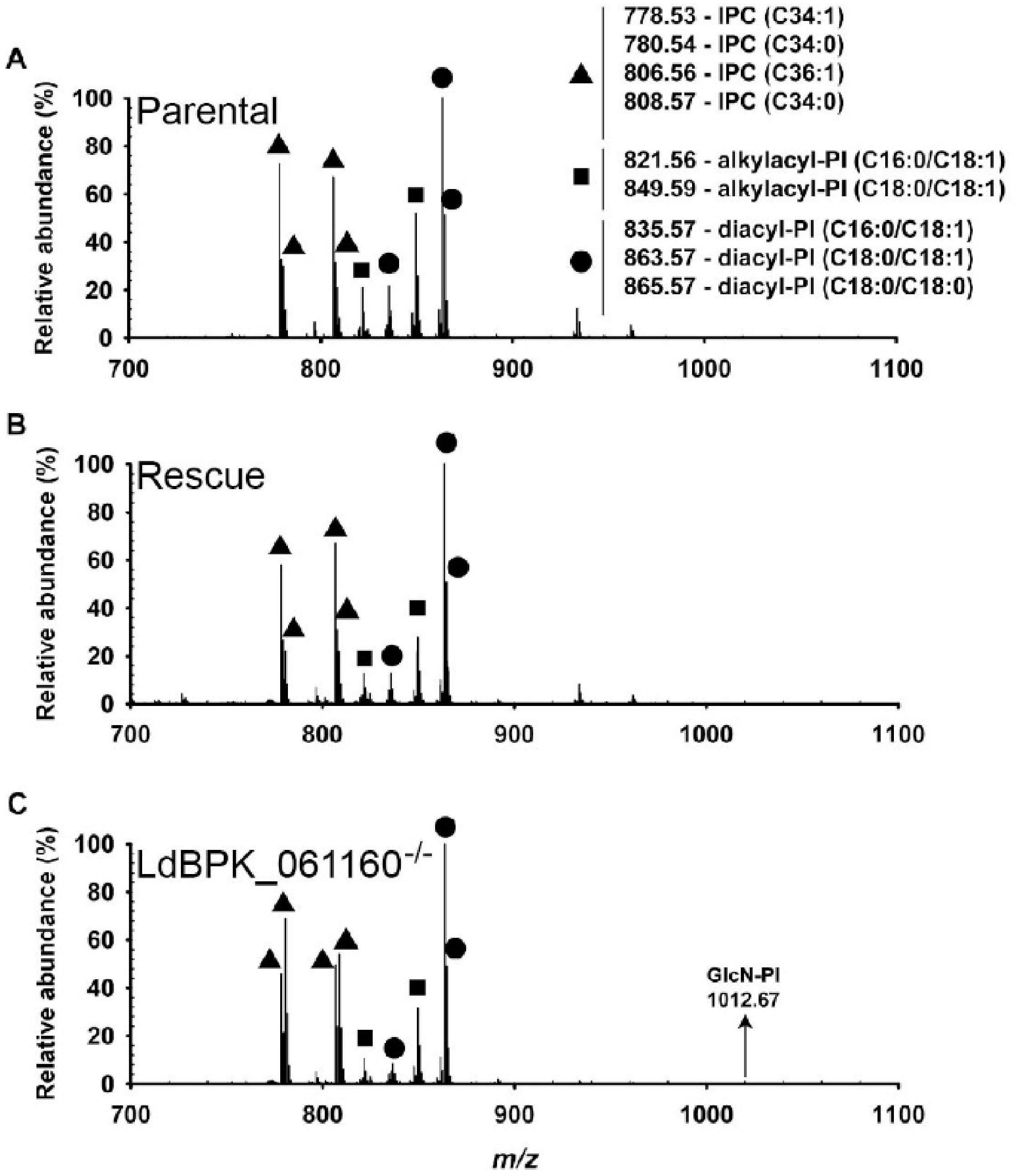

Figure S1. Lipid profiling by negative ion ES-MS identifies the bulk cellular inositol-phospholipids and the GlcN-PI GPI anchor biosynthetic intermediate. Intense $[M\text{-}H]^-$ ions were observed in parental (**A**), genetically rescued **(B)** and *LdBPK_61160* null mutant **(C)** lipid extracts for inositol phosphorylceramide (triangles), diacyl-PI (circles) and alkylacyl-PI (squares) species. The identities of the major ions are indicated in the inset key. The identities of these species were subsequently confirmed by $ES\text{-}MS^2$ (data not shown).
