# Supplementary material for: The Leishmania donovani Ortholog of the Glycosylphosphatidylinositol Anchor Biosynthesis Cofactor PBN1 Is Essential for Host Infection
Source: mBio. 2022 Apr 14;13(3):e00433-22. doi: 10.1128/mbio.00433-22 (PMC9239262; doi:10.1128/mbio.00433-22)
Supplement: FIG S2 [file mbio.00433-22-s0003.pdf]

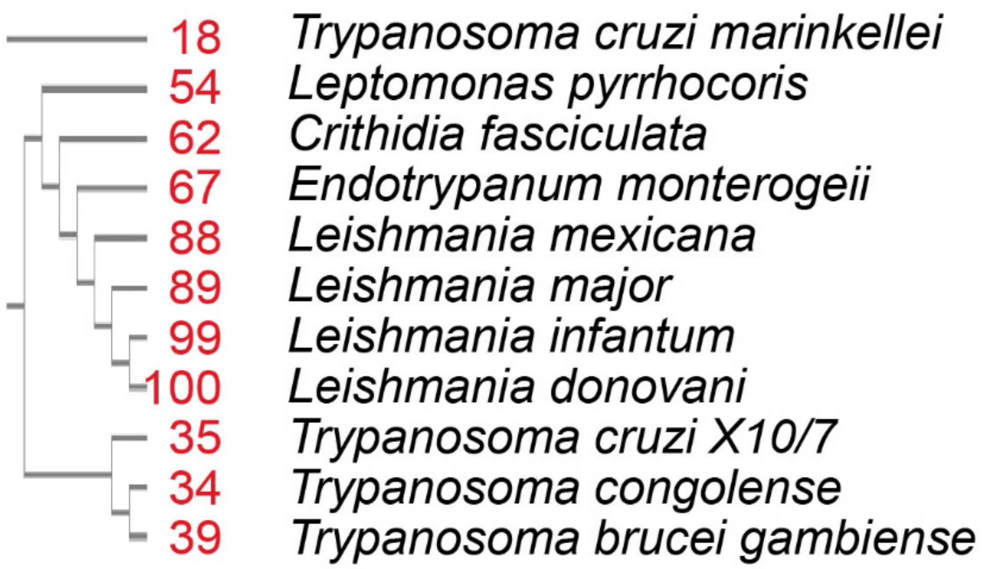

Figure S2. A phylogenetic tree generated from a clustal omega alignment of selected PBN1 homologs from selected parasites. Numbers in red represent the percentage amino acid identity between LdBPK_061160 and each species.
