# Supplementary material for: The Leishmania donovani Ortholog of the Glycosylphosphatidylinositol Anchor Biosynthesis Cofactor PBN1 Is Essential for Host Infection
Source: mBio. 2022 Apr 14;13(3):e00433-22. doi: 10.1128/mbio.00433-22 (PMC9239262; doi:10.1128/mbio.00433-22)
Supplement: FIG S3 [file mbio.00433-22-s0004.pdf]

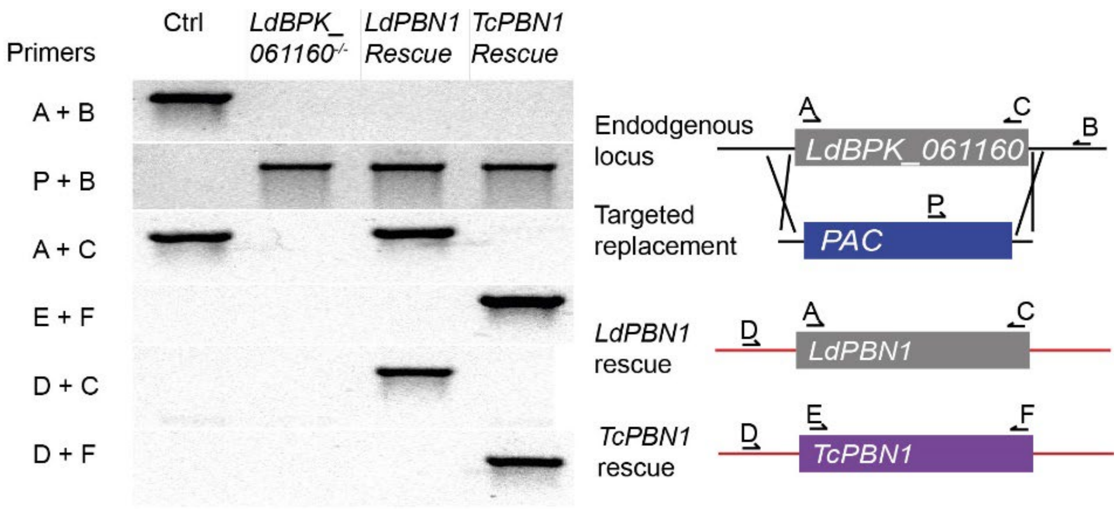

Figure S3. Confirmation of *TcPBN1* rescue genotyping. Diagnostic PCRs (*left panel*) demonstrating the loss of *LdBPK_061160* is due to targeted replacement at the endogenous locus (primers A+B or P+B) and genetic reconstitution with either *LdBPK_061160* (primers A+C or D+C) or *TcPBN1* (Primers E+F or D+F) is specific to a non-endogenous locus. Schematic (*right panel*) of the *LdBPK_061160* endogenous locus and targeted replacement with *PAC* with the *TcPBN1* and *LdPBN1* genetic rescues with associated diagnostic primers.
