# Supplementary material for: The Leishmania donovani Ortholog of the Glycosylphosphatidylinositol Anchor Biosynthesis Cofactor PBN1 Is Essential for Host Infection
Source: mBio. 2022 Apr 14;13(3):e00433-22. doi: 10.1128/mbio.00433-22 (PMC9239262; doi:10.1128/mbio.00433-22)
Supplement: TABLE S1 [file mbio.00433-22-s0001.pdf]

Gene	Species	Percentage identity %
LdCL_060016700	*Leishmania donovani CL-SL*	100
LdBPK_061160.1	*Leishmania donovani BPK282A1*	100
LdBPK.06.2.001160	*Leishmania donovani strain LV9*	100
LINF_060017000	*Leishmania infantum JPCM5*	99.27
LTRL590_060016800	*Leishmania tropica L590*	92.67
LGELEM452_060016300	*Leishmania gerbilli strain LEM452*	92.67
LTULEM423_060017200	*Leishmania turanica strain LEM423*	92.31
LAEL147_000084500	*Leishmania aethiopica L147*	91.58
LARLEM1108_060017100	*Leishmania arabica strain LEM1108*	90.48
LmjF.06.1120	*Leishmania major strain Friedlin*	89.38
LMJSD75_060017900	*Leishmania major strain SD 75.1*	89.38
LMJLV39_060017700	*Leishmania major strain LV39c5*	89.01
LmxM.06.1120	*Leishmania mexicana MHOM/GT/2001/U1103*	87.91
LAMA_000085400	*Leishmania amazonensis MHOM/BR/71973/M2269*	87.55
LtaP06.1100	*Leishmania tarentolae Parrot-TarII*	77.66
LbrM.06.2.001100	*Leishmania braziliensis MHOM/BR/75/M2904 2019*	77.29
LPAL13_060017400	*Leishmania panamensis MHOM/COL/81/L13*	76.19
LPMP_061100	*Leishmania panamensis strain MHOM/PA/94/PSC-1*	76.19
LENLEM3045_060017800	*Leishmania enriettii strain LEM3045*	72.53
LMARLEM2494_060016800	*Leishmania sp. MAR LEM2494*	67.4
EMOLV88_060016400	*Endotrypanum monterogeii strain LV88*	67.16
CFAC1_180030200	*Crithidia fasciculata strain Cf-Cl*	61.88
Lsey_0191_0170	*Leptomonas seymouri ATCC 30220*	57.36
LpyrH10_03_1330	*Leptomonas pyrrhocoris H10*	53.93
Baya_009_0800	*Blechomonas ayalai B08-376*	43.06
Tb927.7.5710	*Trypanosoma brucei brucei TREU927*	38.82
TevSTIB805.7.6260	*Trypanosoma evansi strain STIB 805*	38.82
Tb427_070062900	*Trypanosoma brucei Lister strain 427 2018*	38.82
Tbg972.7.6640	*Trypanosoma brucei gambiense DAL972*	38.82
C4B63_30g269	*Trypanosoma cruzi Dm28c 2018*	37.17
DQ04_04001060	*Trypanosoma grayi ANR4*	36.93
TcCLB.508173.240	*Trypanosoma cruzi CL Brener Non-Esmeraldo-like*	35.92
C3747_54g233	*Trypanosoma cruzi TCC*	35.92
TM35_000541270	*Trypanosoma theileri isolate Edinburgh*	35.8
TcYC6_0045510	*Trypanosoma cruzi Y C6*	35.51
C3747_84g108	*Trypanosoma cruzi TCC*	35.51
C4B63_163g27	*Trypanosoma cruzi Dm28c 2018*	35.1
TcBrA4_0072560	*Trypanosoma cruzi Brazil A4*	34.69
TcX107A1	*Trypanosoma cruzi X10/7*	34.69
TcIL3000.A.H_000541600	*Trypanosoma congolense IL3000 2019*	34.39
TRSC58_05747	*Trypanosoma rangeli SC58*	33.47
TcBrA4_0087100	*Trypanosoma cruzi Brazil A4*	31.75
TvY486_0705834	*Trypanosoma vivax Y486*	31.37
PCON_0006920	*Paratrypanosoma confusum CUL13*	29.89
Tc_MARK_10262	*Trypanosoma cruzi marinkellei strain B7*	18.23

Table S1. Identification of PBN1 homologs in other trypanosomatid parasites. Percentage identity at the amino acid level was calculated from a pairwise comparison of a Clustal omega multiple sequence alignment.
