# Supplementary material for: The Leishmania donovani Ortholog of the Glycosylphosphatidylinositol Anchor Biosynthesis Cofactor PBN1 Is Essential for Host Infection
Source: mBio. 2022 Apr 14;13(3):e00433-22. doi: 10.1128/mbio.00433-22 (PMC9239262; doi:10.1128/mbio.00433-22)
Supplement: FIG S4 [file mbio.00433-22-s0005.pdf]

```
ScPBN1          MVTRHRVTVLYNAPEDIGNHMRQNDTHLTVRGGSGVVLQQRWLLERTGSLDKSFTRITWR    60
LdBPK_061160    -----------------------------MSRVVVM--------ALA---LSFAM---A    16
HsPIG-X         ----------------------------MAARVAAVRAAAWLLLGAA---TGLTR---G    25
                                               .   .:          .    .::

ScPBN1          PRADLARSLSVIENELSAGFSVYSNSSDVPERFITNPVYNSFHSEKFDIEQYLPPEVDLN    120
LdBPK_061160    AA------LAV--TAVQSGSCIF---L-------TSPPSYTFIGG---------------    43
HsPIG-X         PA------AAF--TAARSDAGIRAMCS-------EIILRQEVLKD---------------    55
                         :.  .    :. :                   .

ScPBN1          LSWNPEDFTYDISVEPTQIQIVEYRLLKQGEEFTIARVKDEKLEVGVFFVDASDESDVDI    180
LdBPK_061160    ------------------------------------------------------------    43
HsPIG-X         ------------------------------------------------------------    55

ScPBN1          GGIRCNWRMDDGKMERCQKTSLLYKQGHIAYNHSTTTTSLYLNEPIGLHPKIMIDL---T    237
LdBPK_061160    ----------------------------------------------GFHMQLALEYPLPA    57
HsPIG-X         ----------------------------------------------GFHRDLLIKVKFGE    69
                                                              *:* .: :.

ScPBN1          DFEERPKCMYLMHLQLPLELFIDKFQSSPLLLF---------------------------    270
LdBPK_061160    -------KDVRLSFDLPKSFFVDEAEAEQLYRMEVLSADPMTQGTAAVVVADVTRAYSPL    110
HsPIG-X         SIEDLHTCRLLIKQDIPAGLYVDPYELASLRERNIT-----------------------E    106
                           :  ::*  :::*  :   *

ScPBN1          -----GEDDLELPEYSLRDKAWGSESIFELK---------------A--------GTMNE    302
LdBPK_061160    RMSSRYAFDIEAPVFKVNYTTNHVELTFQQLGGDGAGSALDAYLAEDGAQAQVPFRARLV    170
HsPIG-X         AVMVSENFDIEAPNYLSKE----SEVLI--------------YARRD-SQ--CIDCFQAF    145
                        *:* * :  .      *  :

ScPBN1          VTLHTRYIEPSNNKGDKLEVSFDPEVILACDTGDNKVSRNPFYKK---------GLG-YE    352
LdBPK_061160    IPIHSRYEVLDTKTP--------FSLLRFITGEGAYVRRCLTRVDVAGRADSRCLTGAYR    222
HsPIG-X         LPVHCRYHRPHSEDGEASIVVNNPDLLMFCD-QEFPILKCWAHSEVAAP----CALENE-    199
                : :* **    .:           .::         : :     .

ScPBN1          SLF--TDDTTFRHLNSTTLLVPIPRPDTK--DYSKIKNGTLLCLLISIIYIFSKVFGNNK    408
LdBPK_061160    SSATVAKSEEKPHYK-HCLDLPVGLLADLPYVYHALMGLLVAGAALVILAIR--------    273
HsPIG-X         DICQWNKMKYKSVYKNVILQVPVGLTVHTSLVCSVTLLITILCSTLILVAVF--KYGHFS    257
                .     .        :  * :*:                 :    :  :: :

ScPBN1          KKRSVKRE    416
LdBPK_061160    --------    273
HsPIG-X         L-------    258
```

Figure S4. Alignment of *ScPBN1*, *LdBPK_061160* and *HsPIG-X*. Alignment was generated using Clustal Omega showing conserved amino acids (*) between all three orthologues.
